# Residual Flexural Performance of Epoxy Polymer Concrete under Hygrothermal Conditions and Ultraviolet Aging

**DOI:** 10.3390/ma12213472

**Published:** 2019-10-23

**Authors:** Dongpeng Ma, Zhiwei Pan, Yiping Liu, Zhenyu Jiang, Zejia Liu, Licheng Zhou, Liqun Tang

**Affiliations:** State Key Laboratory of Subtropical Building Science, School of Civil Engineering and Transportation, South China University of Technology, Guangzhou 510640, China; 201610101013@mail.scut.edu.cn (D.M.); scutpanzhiwei@163.com (Z.P.); zjliu@scut.edu.cn (Z.L.); ctlczhou@scut.edu.cn (L.Z.); lqtang@scut.edu.cn (L.T.)

**Keywords:** epoxy polymer concrete, hygrothermal conditions, ultraviolet aging, flexural strength, layered beam model

## Abstract

Epoxy polymer concrete (EPC) has found increasing applications in infrastructure as a rising candidate among civil engineering materials. In most of its service environments, EPC is inevitably exposed to severe weather conditions, e.g., violent changes in temperature, rain, and ultraviolet (UV) radiation. In this paper, we designed an accelerated aging test for EPC, which includes periodic variation of temperature and water spray, as well as intensive UV-light irradiation, imitating the outdoor environment in South China. The experimental results show that the flexural performance of EPC is found deteriorate with the aging time. An aging process equivalent to four years (UV radiation dose) results in up to 8.4% reduction of flexural strength. To explore the mechanisms of observed performance degradation, the EPC specimen in the four-point-bending test is considered as a layered beam. The analysis indicates that the loss of flexural load-carrying capacity of an aged EPC beam is dominated by the reduction of mechanical properties of the surface layer. The mechanical properties of the surface layer are closely associated with the aging of epoxy mortar, which can be approximated as a reciprocal function of the aging time. By introducing damage to the surface layer into the layered beam, the proposed model demonstrates a good ability to predict the residual flexural strength of EPC during the aging process

## 1. Introduction

Epoxy polymer concrete (EPC) is an increasingly popular type of concrete made by mixing epoxy resin, crushed stone, and other fillers in a certain proportion, where resin acts as binder for the other components [1]. Compared to cement concrete, the advantages of EPC include high strength, good fracture resistance and short curing time [2,3,4]. These excellent properties promote its wide applications in civil engineering, where it serves as bridge-deck overlays [5,6], machine tool beds [7,8], repair material [9,10], railway slabs [11] and space applications [12,13], etc.

However, the mechanical properties of epoxy resins and their composites are found to be significantly affected by UV radiation, temperature and water penetration [14,15]. In tropical and subtropical regions, EPC is exposed to strong UV light and high temperatures and humidity, which cause a great challenge to the weatherability of EPC. In recent years, Reis et al. studied the fracture properties of epoxy polymer concrete and fiber-reinforced epoxy polymer concrete under atmospheric [16], marine [17], and temperature conditions [18]. It was found that UV light and temperature lead to substantial degeneration of flexural strength and fracture toughness of EPC. Oussama [12] investigated the mechanical and physical properties of EPC exposed to 250 °C. Their results show a significant loss of strength of EPC due to the thermo-oxidative degradation of the epoxy resin. It was also found that chemical processes, e.g., acid etching, can weaken the mechanical properties of polymer concrete [19,20,21]. Unfortunately, the reported work heretofore is focused on experimental observation, lacking in the exploration of quantitative relationships between the residual performance of EPC and complex environmental factors.

In this paper, an experimental study was carried out systematically on the flexural performance of EPC during an accelerated aging test, which imitates the weather conditions in South China. To analyze the degradation of flexural performance, the aged EPC specimen in the four-point bending test is simplified as a layered beam composed of aged and unaged layers. A mechanical model is proposed to depict the relationship between the ultimate bending moment and characteristics including the thickness of damaged region, the degree of damage and the maximum compressive strain. The capability of the model to predict the residual flexural strength of EPC is verified by the experimental results.

## 2. Specimen Preparation and Testing Program

### 2.1. Materials

Granite aggregate was supplied by Fujian Shiyufa Stone Co. Ltd. The mass percentage of each size of aggregate is shown in Table 1. The aggregate gradation was designed for the purpose of achieving better road performance for pavement materials, and is characterized by a high content of larger aggregate sizes [22]. The gradation has been used in important highway and bridge-paving projects in China. The epoxy binder is made by mixing components bisphenol-A resin (BS5461A) and amine hardener (BS5462) in the mass proportion of 2:1, both of which are supplied by Fuzhou Baisheng Fine Chemicals Pte. Ltd. The Epoxy Equivalent Weight (EEW) and density of the resin is 200 g/equiv. and 1.1 g/cm^3^ respectively. The Amine Hydrogen Equivalent Weight (AHEW) and density of the hardener is 100 g/equiv. and 0.985 g/cm^3^ respectively. The EPC specimens were prepared by adding graded aggregates to the proportional epoxy adhesive, stirring repeatedly for 3 min and filling the mixture into the cuboid mold. The compaction of the EPC specimens was completed by pounding a rubber cushion block placed on the mixture. The density of the specimens was strictly controlled to 2.04 g/cm^3^, which avoids high voids content due to under-compaction or crushed aggregates caused by over-compaction. The size of EPC specimens was 50 mm × 50 mm × 200 mm. After curing for 72 h at 25 °C, the EPC specimens were demolded. In this study, EPC specimens were prepared with a weight percentage of resin of 11.5 wt.%.

### 2.2. The Accelerated Aging Program

**Figure 1:**
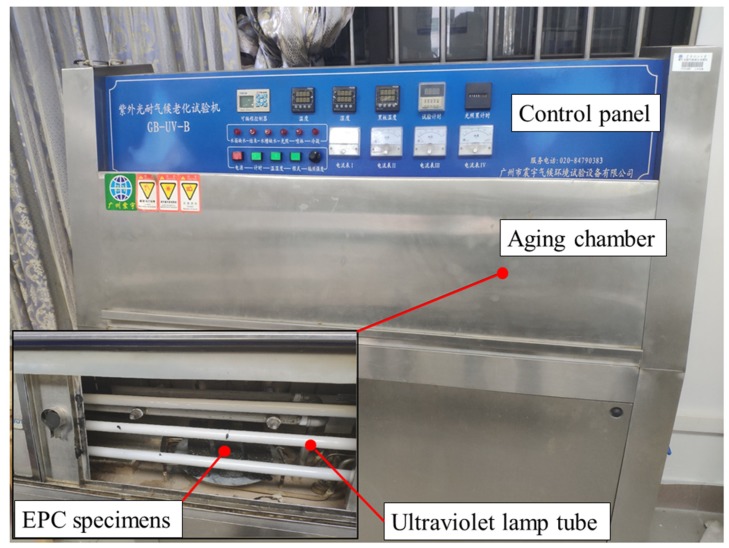
GB-UV-B UV weathering test chamber for accelerated aging of Epoxy Polymer Concrete (EPC).

According to meteorological statistics of Guangzhou city, the average annual UV radiation is 262.4 MJ/m^2^. The accelerated aging test of epoxy polymer concrete was carried out in a GB-UV-B UV weathering test chamber (manufactured by Guangzhou Zhenyu Climate Environment Test Equipment Co., Ltd.), shown as Figure 1. There were 8 lamp tubes in the test chamber, each of which emitted UV power of 12 W, and the average distance between the lamp and the specimen was 70 mm. The UV-light intensity on the surface of the specimen can be calculated through:

(1) $$I_{e}=\frac{12\times 8}{0.07^{2}\times 4\times \pi }=1560\;W/m^{2}$$

The aging test was divided into two alternating processes: ultraviolet radiation and condensation. During the whole process, the temperature alternation of day and night in the real environment was simulated by the temperature cycle. Therefore, the accelerated aging time to simulate aging for one year was 93.3 h, calculated by:

(2) $$t=\frac{262.4\times 10^{6}}{1560}\times 2=3.36\times 10^{5}\;s=93.3\;h$$

**Figure 2:**
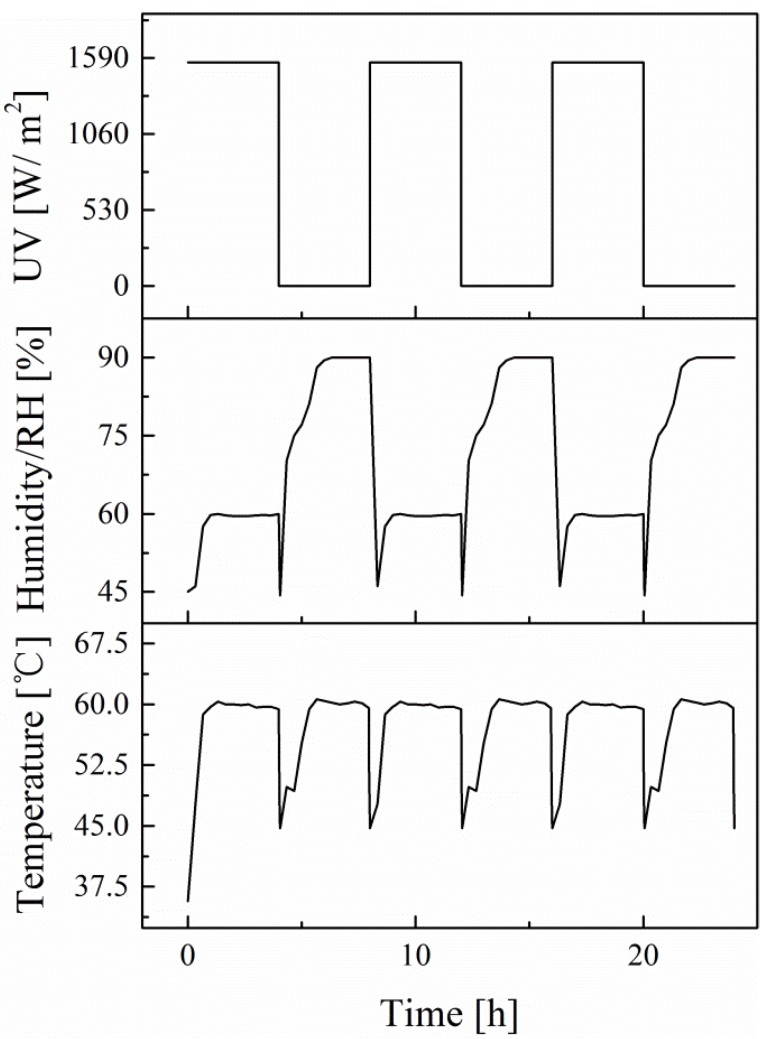
The time-course curves for UV light, humidity, and temperature.

The time course curves for UV light, temperature, and humidity are shown in Figure 2. The equivalent aging time of EPC specimens was 1 year, 2 years and 4 years. The aging scheme was referred to the Standard Practice for Operating Fluorescent Ultraviolet (UV) Lamp Apparatus for Exposure of Nonmetallic Materials (ASTM G 154-16) [23] and each aging cycle was 8 h as shown in Figure 2. The default range of relative humidity (RH) was 60–90%, controlled by the spraying program. The range of RH was chosen to simulate the humidity in South China. The sharp difference between the two humidity platform of 60% and 90% resulted from the decrease in temperature, inevitably facilitating the accumulation of spray and forming drops. The temperature and RH of the laboratory was 25 °C and 70% respectively. At the same time, the blank control group without aging was prepared. For each set of EPC specimens, at least three specimens were tested to eliminate the influence of accidental error.

### 2.3. The Four-Point Bending Test of EPC

**Figure 3:**
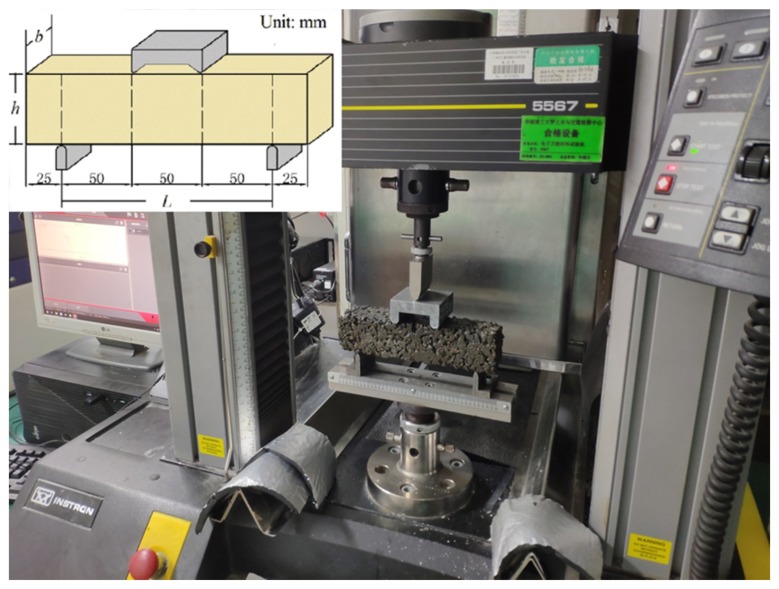
Schematic diagram and photo of the four-point bending test of EPC.

After aging, a four-point bending test was carried out to study the residual flexural performance of the EPC under hygrothermal conditions and ultraviolet aging. The tests were conducted at a velocity 1 mm/min on an Instron 5567 universal tester (Norwood, MA, USA), as shown in Figure 3. The flexural strength of EPC is determined according to:

(3) $$\sigma_{b}=\frac{PL}{bh^{2}}$$

where *P* is the peak load, *L* is the span (*L* = 150 mm), *b* is the width of the specimen (*b* = 50 mm), and *h* is the height of the specimen (*h* = 50 mm). The corresponding ultimate bending moment can be calculated by:

(4) $$M=\frac{PL}{6}$$

### 2.4. Modulus Modification Testing of EPC

**Figure 4:**
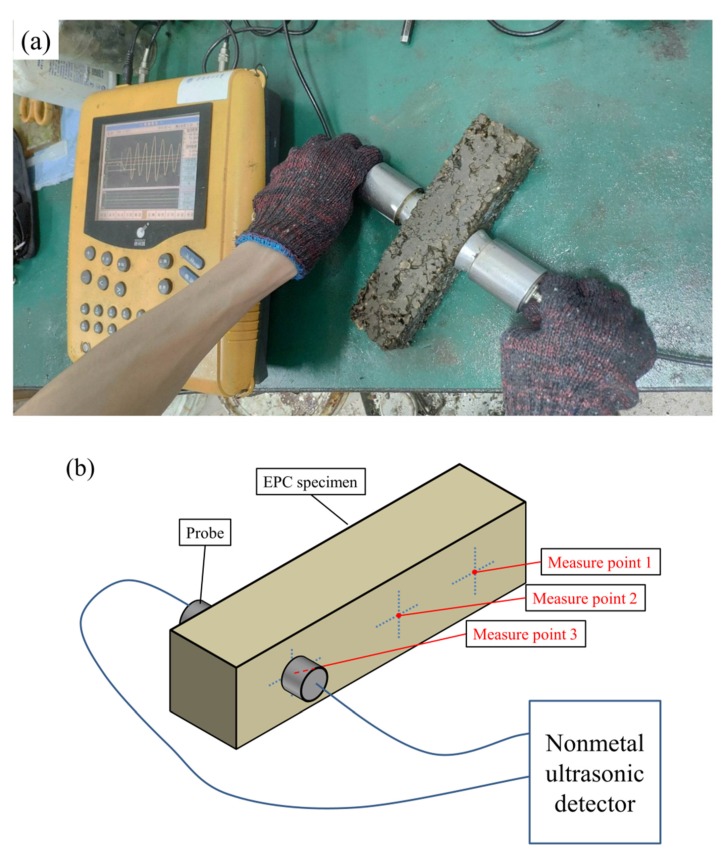
(**a**) The detection of ultrasonic wave velocity propagating through EPC; (**b**) Schematic diagram of the detection.

Usually for specimens with large stiffness, the elastic modulus obtained directly from the universal tester is inaccurate due to the inevitable deformation of the universal tester itself. Therefore, a modulus modification was necessary for EPC. Here, a nonmetal ultrasonic detector was used to determine the elastic modulus of EPC. By measuring the ultrasonic propagation velocity in nonmetallic materials, the elastic modulus of EPC can be expressed as:

(5) $$E=v^{2}\rho$$

where ρ is the density of the material ($\rho =2.04\;g/{\mathrm{cm}}^{3}$). The velocity of ultrasonic waves propagating through EPC v was measured as shown in Figure 4a. For each specimen, three measurement points along its length were selected for the detection to eliminate the influence of accidental error, as shown in Figure 4b.

### 2.5. Three-Point Bending Test of Epoxy Mortar

**Figure 5:**
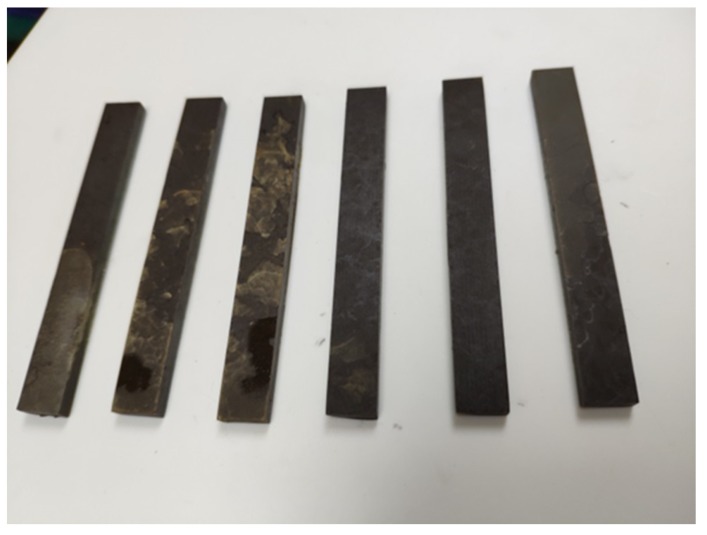
Epoxy mortar specimens after accelerated aging.

**Figure 6:**
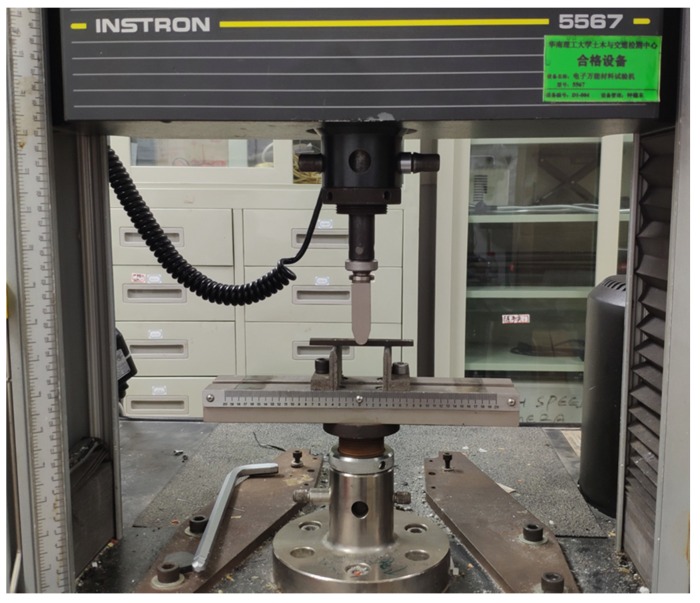
Three-point bending test of epoxy mortar specimens.

Epoxy mortar was fabricated with the same ingredients as EPC, except only aggregates smaller than 0.3 mm were added into the resin. The size of each epoxy mortar specimen was 10 mm × 4 mm × 80 mm. The epoxy mortar specimens were cured for 72 h at 25 °C before demolding. Then the accelerated aging test was conducted following the same aging program presented in Section 2.2. After aging, the specimens were taken out and the three-point bending test was carried out, as shown in Figure 5 and Figure 6. The span of each three-point bending specimen was 40 mm. For each set of epoxy mortar specimens, at least three specimens were tested to eliminate the influence of accidental error.

## 3. Experimental Results

The velocity of ultrasonic waves propagating through EPC was 3.66 ± 0.09 km/s (mean value with standard deviation), read directly from the nonmetal ultrasonic detector. Accordingly, the elastic modulus obtained from the measurement was 27.4 ± 1.4 GPa, calculated by Equation (5). The elastic modulus was used to modify the stress–strain curves in the four-point bending test of EPC.

**Figure 7:**
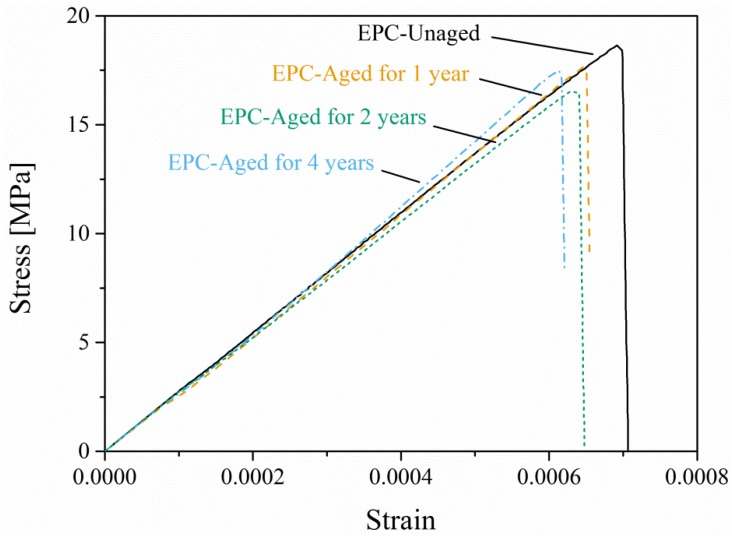
Stress–strain curves of EPC after various accelerated aging times under four-point bending.

Figure 7 shows the stress–strain curves of EPC after various accelerated aging times. It can be observed that after different aging times, EPC maintains the characteristic of brittle failure. All the curves grow linearly with the deflection during the loading process, and decline sharply after reaching the maximum value. The fracture positions were all located in the pure bending section of the specimens. With increased aging time, the flexural strength of EPC decreased obviously. After aging for four years, the flexural strength of EPC decreased by 8.4%. The flexural strength and the corresponding ultimate bending moment (mean value with standard deviation) are listed in Table 2.

For polymer concrete, the complexity of its components leads to the divergence of the flexural strength, which can be found in other authors’ research [12,19]. Moreover, complicated environmental factors may further enlarge the divergence of the results. Therefore, the results of the four-point bending test of EPC in Table 2 are reasonable.

**Figure 8:**
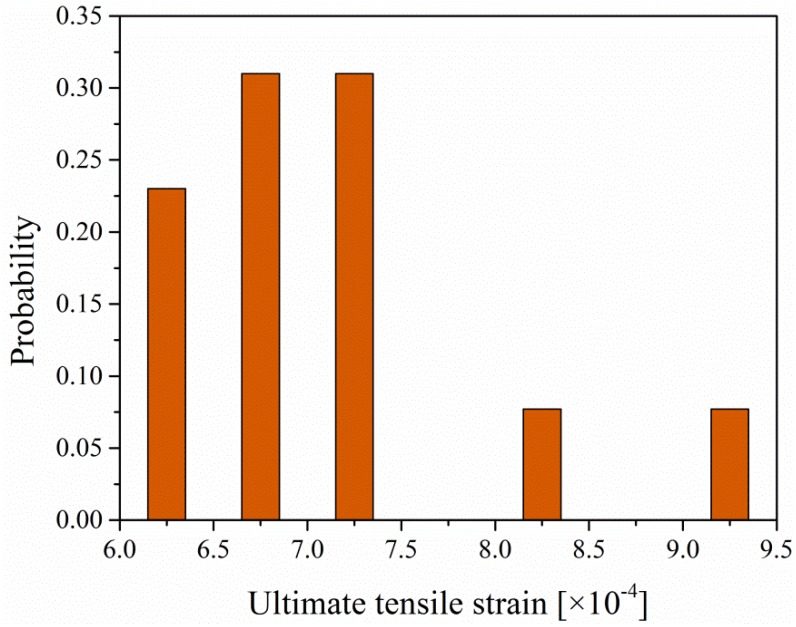
Statistics of the ultimate strain of EPC.

It can be seen from Figure 7 that the ultimate strain of EPC after aging is very close to that of the unaged ones. The statistics of the ultimate strain of the EPC specimens tested in Section 2.3 further support this statement. Figure 8 shows the probability of the ultimate strain and it is found that the ultimate strain is distributed mainly in the interval 0.000625–0.000725. It is supposed that once the tensile strain exceeds the ultimate value, cracks will occur and the specimen will lose its load-carrying capacity. Since there is a slight difference of the tensile ultimate strain between the unaged and aged EPC, the ultimate tensile strain $\varepsilon_{u}$ can be uniformly calculated by:

(6) $$\varepsilon_{t}=\varepsilon_{u}=\frac{f}{E}=\frac{18.06\;\mathrm{MPa}}{27.4\;\mathrm{GPa}}=0.00066$$

where f is the flexural strength of unaged EPC.

**Figure 9:**
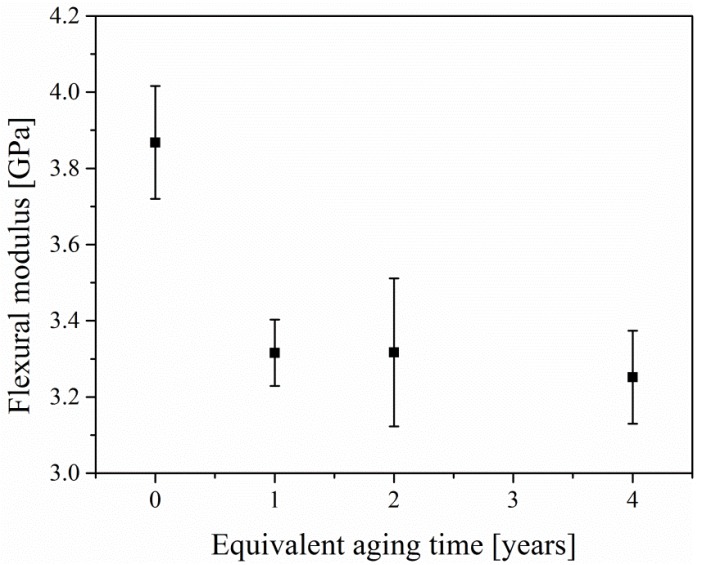
Flexural modulus of epoxy mortar at different aging times.

Figure 9 shows the flexural modulus with various equivalent aging times. It can be seen from the figure that the flexural modulus of epoxy mortar descends sharply after aging. Then, with the increase of aging time, the flexural modulus of epoxy mortar decreases slowly. The reason for this change trend is that UV light can easily enter the interior of the specimen at the initial stage of aging, and afterwards it becomes more and more difficult for UV light to penetrate the deeper part of epoxy mortar.

## 4. Aging Damage Model

**Figure 10:**
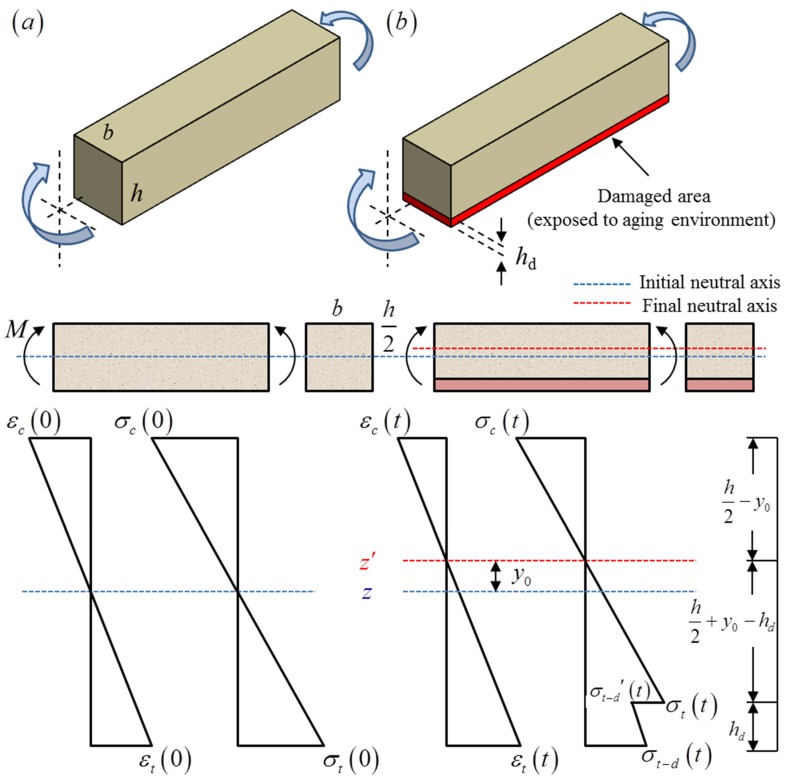
Schematic diagram of loading and stress distribution: (**a**) without considering the aged layer; (**b**) considering the aged layer.

To explore the mechanism of flexural performance degradation of EPC, a layered beam composed of aged and unaged layers was presented as a simplified model. Since only the upper surface of the concrete is directly exposed to UV-light and spray during the aging test, it is reasonable to assume that the damaged area, which we defined as the aged layer, appears only on the upper part of the specimen. Accordingly, the remaining part was defined as the unaged layer. To effectively reveal the impact of aging on the flexural properties of EPC, the aged layer was placed on the tensile side during the four-point bending test. Figure 10 is the schematic diagram of loading and stress distribution obtained correspondingly. Without considering the aged layer, the neutral axis z is located in the middle of the longitudinal section of the specimen based on the assumption of the plane section, as shown in Figure 10a. When the aged layer is taken into consideration, the neutral axis $z^{\prime }$ will deviate from the middle of the longitudinal section and shift towards the compression side of the specimen, as shown in Figure 10b. The offset of the neutral axis is defined as $y_{0}$. Then, the resultant force in the compression area can be expressed as:

(7) $$N_{c}=\frac{1}{2}b\left(\frac{h}{2}-y_{0}\right)\sigma_{c}$$

where $\sigma_{c}$ is the maximum stress on the compressive side of specimen under bending load. The distance between the resultant force in the compression area and the neutral axis $z^{\prime }$ is $y_{c}$, which can be written as:

(8) $$y_{c}=\frac{2}{3}\left(\frac{h}{2}-y_{0}\right)$$

Due to the existence of the aged layer, the stress on the tensile side will appear a step-change between the aged and unaged layer, while the strain maintains continuity. The resultant force in tension area above the aged layer can be expressed as:

(9) $$N_{t}=\frac{1}{2}b\left(\frac{h}{2}-h_{d}+y_{0}\right)\sigma_{t}$$

in which $\sigma_{t}$ is the maximum stress on tensile side of specimen in the unaged layer and $h_{d}$ is the thickness of the aged layer. Accordingly, the distance between $N_{t}$ and the neutral axis $z^{\prime }$ can be calculated by:

(10) $$y_{t}=\frac{2}{3}\left(\frac{h}{2}-h_{d}+y_{0}\right)$$

The resultant force of the aged layer can be written as:

(11) $$N_{t}^{\prime }=\frac{1}{2}bh_{d}\left({\sigma_{t-d}}^{\prime }+\sigma_{t-d}\right)$$

in which ${\sigma_{t-d}}^{\prime }$ is the tensile stress of the aged layer at the step, and $\sigma_{t-d}$ is the maximum tensile stress of the aged layer. The distance between $N_{t}^{\prime }$ and the neutral axis $z^{\prime }$ can be calculated by:

(12) $$y_{t}^{\prime }=\left(\frac{h}{2}-h_{d}+y_{0}\right)+h_{d}\frac{{\sigma_{t-d}}^{\prime }+2\sigma_{t-d}}{3\left({\sigma_{t-d}}^{\prime }+\sigma_{t-d}\right)}$$

Therefore, the equilibrium equations require:

(13) $$\{\begin{array}{c} N_{t}+N_{t}^{\prime }-N_{c}=0\\ N_{t}y_{t}+N_{t}^{\prime }y_{t}^{\prime }+N_{c}y_{c}=M \end{array}$$

where M is the ultimate bending moment obtained from the experiment.

It is assumed that the tensile and compressive properties of the unaged layer satisfy the linear elasticity, while the effect of aging damage is considered in the constitutive relationship of the aged layer. The constitutive relations of the two materials are listed in Equation (14):

(14) $$\{\begin{array}{c} \sigma_{c}=E\varepsilon_{c}\\ \sigma_{t}=E\varepsilon_{t}\\ {\sigma_{t-d}}^{\prime }=\left(1-D\right)E\varepsilon_{t}\\ \sigma_{t-d}=\left(1-D\right)E\varepsilon_{t-d} \end{array}$$

where *D* is the damage factor of the aged layer and defined as the attenuation of modulus, $\varepsilon_{c}$ is the maximum compressive strain of the specimen, while $\varepsilon_{t}$ and $\varepsilon_{t-d}$ is the maximum tensile strain of the unaged and aged layer respectively. Based on the assumption of the plane section, the strain maintains continuity. Therefore $\varepsilon_{c}$, $\varepsilon_{t}$ and $\varepsilon_{t-d}$ satisfy:

(15) $$\{\begin{array}{c} \frac{\varepsilon_{t}}{\varepsilon_{c}}=\frac{\frac{h}{2}-h_{d}+y_{0}}{\frac{h}{2}-y_{0}}\\ \frac{\varepsilon_{t-d}}{\varepsilon_{c}}=\frac{\frac{h}{2}+y_{0}}{\frac{h}{2}-y_{0}} \end{array}$$

In fact, based on the above derivation, the maximum tensile strain of the aged layer will exceed the ultimate tensile strain of unaged EPC, which is unreasonable. Therefore, we assume that when the local tensile strain of the aged layer exceeds $\varepsilon_{u}$, the corresponding area of the aged layer will crack and cannot continue to bear load during the tensile process. With this assumption, Equations (11) and (12) are modified to:

(16) $$N_{t}^{\prime }=\frac{1}{2}b\left(\frac{\sigma_{t-d}\left(\frac{h}{2}-y_{0}\right)}{\left(1-D\right)E\varepsilon_{c}}-\left(\frac{h}{2}-h_{d}+y_{0}\right)\right)\left({\sigma_{t-d}}^{\prime }+\sigma_{t-d}\right)$$

(17) $$y_{t}^{\prime }=\left(\frac{h}{2}-h_{d}+y_{0}\right)+\left(\frac{\sigma_{t-d}\left(\frac{h}{2}-y_{0}\right)}{\left(1-D\right)E\varepsilon_{c}}-\left(\frac{h}{2}-h_{d}+y_{0}\right)\right)\frac{{\sigma_{t-d}}^{\prime }+2\sigma_{t-d}}{3\left({\sigma_{t-d}}^{\prime }+\sigma_{t-d}\right)}$$

Furthermore, according to the assumption based on the experimental results in Section 3, the maximum tensile strain of the aged layer $\varepsilon_{t-d}$ should also be modified to $\varepsilon_{u}$ in Equation (15).

Finally, a function of ultimate bending moment M, D, and $h_{d}$ can be obtained as follows

(18) $$\begin{array}{cc} M & =\frac{1}{3\left(\frac{h}{2}-y_{0}\right)}bE{\left(\frac{h}{2}-h_{d}+y_{0}\right)}^{3}\varepsilon_{c}+\frac{1}{2}b\left(\frac{\varepsilon_{u}\left(\frac{h}{2}-y_{0}\right)}{\varepsilon_{c}}-\left(\frac{h}{2}-h_{d}+y_{0}\right)\right)\left(1-D\right)E\left(\frac{\frac{h}{2}-h_{d}+y_{0}}{\frac{h}{2}-y_{0}}\varepsilon_{c}+\varepsilon_{u}\right)\\ & \times \left[\left(\frac{h}{2}-h_{d}+y_{0}\right)+\left(\frac{\varepsilon_{u}\left(\frac{h}{2}-y_{0}\right)}{\varepsilon_{c}}-\left(\frac{h}{2}-h_{d}+y_{0}\right)\right)\times \left(\frac{\frac{\frac{h}{2}-h_{d}+y_{0}}{\frac{h}{2}-y_{0}}\varepsilon_{c}+2\varepsilon_{u}}{3\left(\frac{\frac{h}{2}-h_{d}+y_{0}}{\frac{h}{2}-y_{0}}\varepsilon_{c}+\varepsilon_{u}\right)}\right)\right]+\frac{1}{3}bE{\left(\frac{h}{2}-y_{0}\right)}^{2}\varepsilon_{c} \end{array}$$

where:

(19) $$y_{0}=\frac{D\left(h-h_{d}\right)h_{d}}{2\left(h-h_{d}D\right)}$$

## 5. Parameter Calibration and Verification

### 5.1. Calibration of the Damage Factor

Since granite is not sensitive to aging conditions, we used the degradation of flexural modulus of epoxy mortar after aging to define the damage factor of the aged layer.

**Figure 11:**
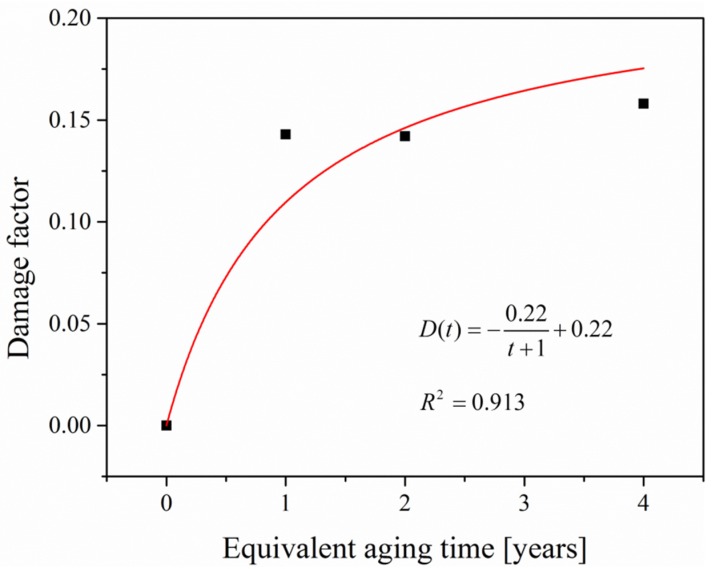
Damage factor–equivalent aging time curve.

The damage factor is defined by the ratio of the attenuation to the modulus of unaged epoxy mortar (i.e., aging for 0 years), and the relationship between the damage factor and aging time can be expressed as a reciprocal function by curve fitting, shown as Equation (20) and Figure 11. The Relative Root Mean Square Error (RRMSE) of the fitting curve is 0.17, implying a good imitation of the damage evolution.

(20) $$D\left(t\right)=-\frac{0.22}{t+1}+0.22$$

### 5.2. Calibration of the Maximum Compressive Strain

Based on the fact that the compressive side of concrete is almost unaffected by aging, the elastic modulus of the compressive side is assumed to be constant. Therefore, due to the deviation of the neutral axis after aging, the ratio of the maximum compressive strain of the aged specimen to the ultimate tensile strain of the unaged specimen satisfies the following relationship:

(21) $$\frac{\varepsilon_{c}}{\varepsilon_{t}}=\frac{0.5h-y_{0}}{0.5h}$$

where $\varepsilon_{t}$ is equal to $\varepsilon_{u}$. Therefore, the maximum compressive strain-equivalent aging time curve can be obtained once $y_{0}$ is determined.

### 5.3. Calibration of the Thickness of The Aged Layer

Since the components of EPC are relatively complex, it is difficult to obtain reliable values of aged layer thickness by using composition analysis methods, e.g., X-ray spectroscopy or mechanical analysis methods, e.g., surface scraping. Based on the analysis in Section 5.1, it can be shown that the damage increases sharply in the first year of aging, and then the rate of increase slows down. Therefore, we consider using a function similar to the damage evolution equation to define the evolution process of aging thickness. Then the evolution equation of aged layer thickness can be expressed as:

(22) $$h_{d}\left(t\right)=-\frac{\gamma }{t+1}+\gamma$$

where γ is a parameter determined by experimental result of EPC aged for one year. 

When t=1 year, the parameters mentioned above can be expressed as

▪ D(1)=0.11
▪ $h_{d}\left(1\right)=\frac{\gamma }{2}m$
▪ $y_{0}\left(1\right)=\frac{0.0275\left(0.1-\gamma \right)\gamma }{0.1-0.11\gamma }\;m$
▪ $\varepsilon_{c}\left(1\right)=\frac{0.5\times 0.05-\frac{0.0275\left(0.1-\gamma \right)\gamma }{0.1-0.11\gamma }}{0.5\times 0.05}\times \frac{18.06}{27.4\times 10^{3}}$.

Combined with Equation (18) and based on the ultimate bending moment of EPC aging for one year, we can get γ = 0.0113. Therefore:

(23) $$h_{d}\left(t\right)=-\frac{0.0113}{t+1}+0.0113$$

**Figure 12:**
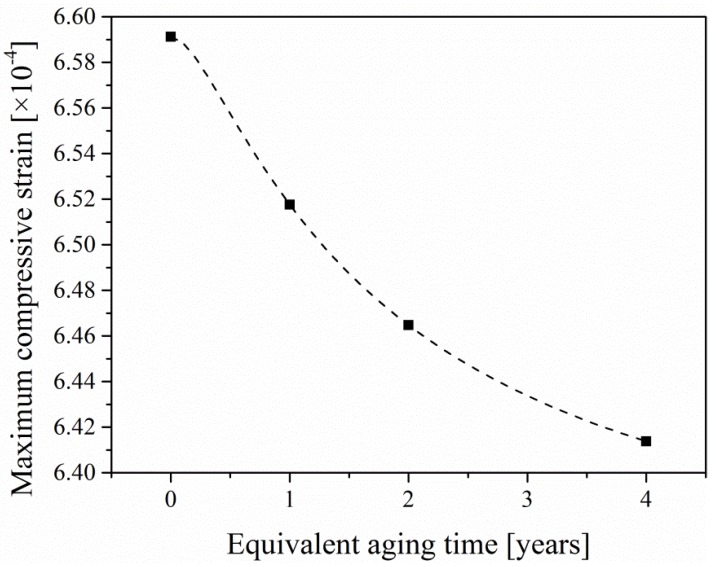
The maximum compressive strain–equivalent aging time curve.

**Figure 13:**
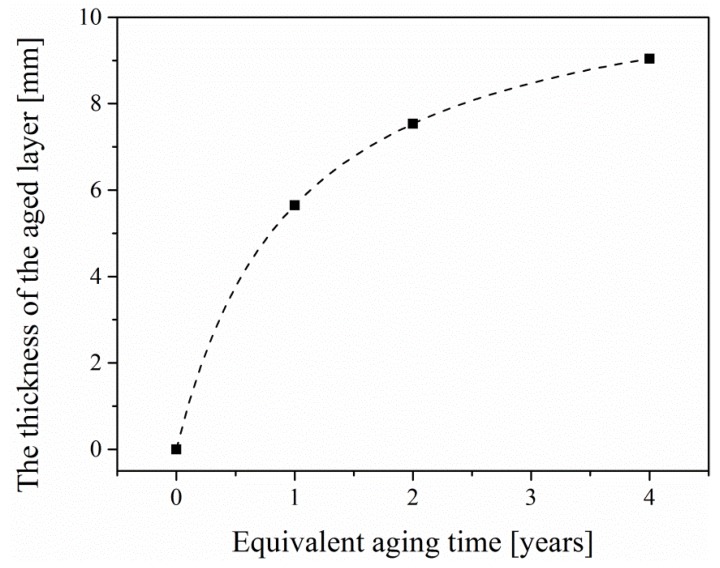
The thickness of the aged layer–equivalent aging time curve.

The maximum compressive strain-equivalent aging time curve mentioned in Section 5.2 can be obtained, as shown in Figure 12. The thickness of the aged-layer-equivalent aging time curve is shown in Figure 13.

### 5.4. Model Verification

**Figure 14:**
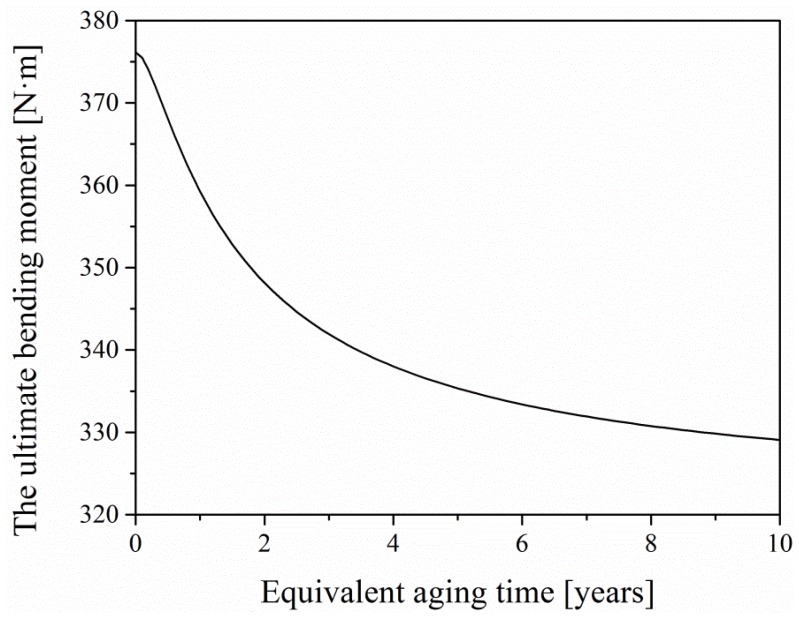
The ultimate bending moment–equivalent aging time curve.

Considering the insensitivity of aggregate to the aging factors, the thickness and damage of the aged layer of EPC are characterized by the analysis performed on epoxy mortar. In order to verify the rationality of the model, the theoretical values of the ultimate bending moment of EPC after aging for two years and four years are obtained by Equation (18). The theoretical values obtained from the model proposed in this study and the corresponding experimental values obtained from the four-points bending test of EPC are listed in Table 3. It can be found that the model can accurately predicts the flexural properties of EPC after aging for two and four years. Moreover, based on the ultimate bending moment model, the flexural properties of EPC after aging for several years can be described as the curve in Figure 14. It can be seen from the graph that the attenuation rate of flexural properties of EPC will gradually slow down after aging, resulting from the limited permeability of the environmental factors.

## 6. Conclusions

The residual flexural performance of epoxy polymer concrete (EPC) under hygrothermal conditions and ultraviolet aging were studied in this paper. Through the experimental study and theoretical analysis, the following conclusions can be obtained:

1. Under the accelerated aging conditions simulating the outdoor environment in South China, the flexural performance of EPC is found to decline with increased aging time. The flexural strength of EPC decreased by 8.4% after aging for four years. After different aging times, EPC maintains the characteristic of brittle failure.
2. A layered beam model is proposed to explore the mechanisms of flexural property degeneration of EPC. The model is based on assumptions that the stiffness of the surface layer is reduced after aging, following the classical damage model. The damaged surface layer loses its load-carrying capacity when its strain exceeds the ultimate tensile strain. The model can accurately predict the performance of EPC after aging of two and four years.
3. The ultimate bending moment model shows that the deterioration of EPC performance will slow down in the subsequent aging process. The aging effects may reach a saturation level in case of enough aging time.

## Figures and Tables

**Table 1:** The mass percentage of aggregate.

Aggregate Size [mm]	<0.3	0.3~0.6	0.6~1.18	1.18~2.36	2.36~4.75	4.75~9.5
Mass Percentage [%]	13.5	5.2	4.8	9.1	13.9	53.5

**Table 2:** Flexural strength and ultimate bending moment of EPC after various accelerated aging times.

Equivalent Aging Time [Years]	0	1	2	4
**Flexural Strength [MPa]**	18.06 ± 1.27	17.24 ± 1.19	16.85 ± 1.61	16.55 ± 0.14
**Ultimate Bending Moment [N·m]**	376.3 ± 26.5	359.2 ± 24.8	351.0 ± 33.5	344.8 ± 2.9

**Table 3:** The theoretical and experimental values of the ultimate bending moment.

Equivalent Aging Time (Years)	Theoretical Values (N·m)	Experimental Values (N·m)	Error (%)
2	348.1	351.0	0.8
4	338.0	344.8	2.0

## References

[1] Bedi R., Chandra R., Singh S.P. (2013). Mechanical properties of polymer concrete. J. Compos..

[2] Davydov S.S., Solomatov V.I., Shvidko Y.I. (1970). Epoxy polymer concrete. Hydrotech. Constr..

[3] Reis J.M.L., Ferreira A.J.M. (2004). A contribution to the study of the fracture energy of polymer concrete and fibre reinforced polymer concrete. Polym. Test..

[4] Ma D., Liu Y., Zhang N., Jiang Z., Tang L., Xi H. (2017). Micromechanical modeling of flexural strength for epoxy polymer concrete. Int. J. Appl. Mech..

[5] Mo L.T., Fang X., Yan D.P., Huurman M., Wu S.P. (2012). Investigation of mechanical properties of thin epoxy polymer overlay materials upon orthotropic steel bridge decks. Constr. Build. Mater..

[6] Wang J., Dai Q., Guo S., Si R. (2019). Mechanical and durability performance evaluation of crumb rubber-modified epoxy polymer concrete overlays. Constr. Build. Mater..

[7] Kim H.S., Park K.Y., Lee D.G. (1995). A study on the epoxy resin concrete for the ultra-precision machine tool bed. J. Mater. Process. Technol..

[8] Cho S.-K., Kim H.-J., Chang S.-H. (2011). The application of polymer composites to the table-top machine tool components for higher stiffness and reduced weight. Compos. Struct..

[9] Yemam D.M., Kim B.-J., Moon J.-Y., Yi C. (2017). Mechanical properties of epoxy resin mortar with sand washing waste as filler. Materials.

[10] Roh I.-T., Jung K.-C., Chang S.-H., Cho Y.-H. (2015). Characterization of compliant polymer concretes for rapid repair of runways. Constr. Build. Mater..

[11] Jeon E.-B., Ahn S., Lee I.-G., Koh H.-I., Park J., Kim H.-S. (2015). Investigation of mechanical/dynamic properties of carbon fiber reinforced polymer concrete for low noise railway slab. Compos. Struct..

[12] Oussama E., Elhem G., Valérie M., Mongi B.O. (2012). Mechanical and physical properties of epoxy polymer concrete after exposure to temperatures up to 250 °C. Constr. Build. Mater..

[13] Naser M.Z., Chehab A.I. (2018). Materials and design concepts for space-resilient structures. Prog. Aerosp. Sci..

[14] Cavasin M., Sangermano M., Thomson B., Giannis S. (2019). Exposure of glass fiber reinforced polymer composites in seawater and the effect on their physical performance. Materials.

[15] Nikafshar S., Zabihi O., Ahmadi M., Mirmohseni A., Taseidifar M., Naebe M. (2017). The effects of UV light on the chemical and mechanical properties of a transparent epoxy-diamine system in the presence of an organic UV absorber. Materials.

[16] Reis J.M.L., Ferreira A.J.M. (2006). The effects of atmospheric exposure on the fracture properties of polymer concrete. Build. Environ..

[17] Reis J.M.L., Ferreira A.J.M. (2005). Effect of marine exposure on fracture properties of epoxy concretes. Polym. Test..

[18] Reis J.M.L., Carvalho A.R., Mattos H.S.d.C. (2014). Effects of displacement rate and temperature on the fracture properties of polymer mortars. Constr. Build. Mater..

[19] Shen Y., Liu B., Lv J., Shen M. (2019). Mechanical properties and resistance to acid corrosion of polymer concrete incorporating ceramsite, fly ash and glass fibers. Materials.

[20] Reis J.M.L. (2010). Fracture assessment of polymer concrete in chemical degradation solutions. Constr. Build. Mater..

[21] Reis J.M.L. (2009). Mechanical characterization of polymer mortars exposed to degradation solutions. Constr. Build. Mater..

[22] Zhang X., Zhang S., Xu W., Huang Z., Liang W., Yuan M. (2012). Application performance-based design of epoxy asphalt concrete applied to steel bridge deck pavement. J. South. China Univ. Technol..

[23] ASTM G 154-16 (2016). Standard Practice for Operating Fluorescent Ultraviolet (UV) Lamp Apparatus for Exposure of Nonmetallic Materials.

